# The Drug Derived Complexity Index (DDCI) Predicts Mortality, Unplanned Hospitalization and Hospital Readmissions at the Population Level

**DOI:** 10.1371/journal.pone.0149203

**Published:** 2016-02-19

**Authors:** Fabio Robusto, Vito Lepore, Antonio D'Ettorre, Giuseppe Lucisano, Giorgia De Berardis, Lucia Bisceglia, Gianni Tognoni, Antonio Nicolucci

**Affiliations:** 1 Department of Clinical Pharmacology and Epidemiology, Mario Negri Sud Foundation, Santa Maria Imbaro (CH), Italy; 2 Regional Health Agency of Puglia, Bari (BA), Italy; 3 Center for Outcomes Research and clinical Epidemiology, Pescara (PE), Italy; 4 IRRCS Mario Negri Milano, Milano (MI), Italy; Zhongshan Hospital Fudan University, CHINA

## Abstract

**Objective:**

to develop and validate the Drug Derived Complexity Index (DDCI), a predictive model derived from drug prescriptions able to stratify the general population according to the risk of death, unplanned hospital admission, and readmission, and to compare the new predictive index with the Charlson Comorbidity Index (CCI).

**Design:**

Population-based cohort study, using a record-linkage analysis of prescription databases, hospital discharge records, and the civil registry. The predictive model was developed based on prescription patterns indicative of chronic diseases, using a random sample of 50% of the population. Multivariate Cox proportional hazards regression was used to assess weights of different prescription patterns and drug classes. The predictive properties of the DDCI were confirmed in the validation cohort, represented by the other half of the population. The performance of DDCI was compared to the CCI in terms of calibration, discrimination and reclassification.

**Setting:**

6 local health authorities with 2.0 million citizens aged 40 years or above.

**Results:**

One year and overall mortality rates, unplanned hospitalization rates and hospital readmission rates progressively increased with increasing DDCI score. In the overall population, the model including age, gender and DDCI showed a high performance. DDCI predicted 1-year mortality, overall mortality and unplanned hospitalization with an accuracy of 0.851, 0.835, and 0.584, respectively. If compared to CCI, DDCI showed discrimination and reclassification properties very similar to the CCI, and improved prediction when used in combination with the CCI.

**Conclusions and Relevance:**

DDCI is a reliable prognostic index, able to stratify the entire population into homogeneous risk groups. DDCI can represent an useful tool for risk-adjustment, policy planning, and the identification of patients needing a focused approach in everyday practice.

## What is new?

Administrative health databases can be used to obtain algorithms useful to forecast readmission and reduce in-care cost. Validated comorbidity indexes (such as Charlson Comorbility Index) have been applied on hospitalization data to predict the risk of death or readmission, but these models do not permit to define the out-patient risk profile. More complex predictive models were obtained to overcome this limitation through the integration of several data-sources, including outpatients, accident and emergency, electronic clinical data from general practitioners, socio-economic data, and community dispensed prescriptions. Unfortunately, the different data-bases required are not always available and/or standardized. Our data show that a much simpler scoring system, solely based on drug prescriptions, can accurately predict one-year and long-term mortality, as well as the risk of unplanned hospitalization and hospital readmission.

## Background

Healthcare utilization, unnecessary care and health care spending increase linearly with the number of chronic conditions affecting an individual. In U.S., 25% of the population with multiple chronic conditions account for two-thirds of total health care spending[1,2].

An accurate prediction of the risk of poor outcomes in individuals with multiple comorbidities would allow health care professionals to focus on patients who are at highest risk of hospital readmissions, inappropriate care, elevated healthcare costs, and mortality. Stratifying patients according to risk can help identifying individuals candidate to an appropriate intervention in order to improve health outcomes, allocate resources more efficiently, reduce costs and facilitate better planning. As an example, several studies have shown that focused care after discharge can decrease the risk of readmission to hospital[3–8]. Several predictive models have been developed, mainly based on clinical, hospital discharge data, or validated comorbidity indexes[7,9,10]. The main limitation of these tools is represented by the difficulty to apply them at the population level, and not only to individuals admitted in hospital or undergoing ad hoc assessments.

An alternative approach can be represented by the use of drug prescription data, using the chronic use of specific classes of drugs as a proxy of chronic diseases and an expression of healthcare complexity. The possibility to use prescription data as indicators of underlying diseases was experienced in many clinical contexts[11–15], and their use to define the clinical risk profile represents the evolution of this process.

## Objective

The aim of the study was to develop and validate the Drug Derived Complexity Index (DDCI), a predictive model derived from drug prescriptions. In particular, we evaluated whether DDCI was able to stratify the general population according to the risk of death, unplanned hospital admission, and readmission, and compared it with the Charlson Comorbidity Index in terms of discrimination and reclassification.

## Research design and methods

We conducted a population-based retrospective cohort study, using a record-linkage analysis of prescription databases, hospital discharge records, and the civil registry, including data on the population aged 40 years or over of the Puglia region in Italy (approximately 2 million out 4.1 citizens in 6 local health authorities).

### Data sources

All Italian citizens have equal access to health care services and are cared for by a general practitioner as part of the National Health System (NHS). With the only exception of some drugs delivered directly by hospital pharmacy (biological agents, some anticancer drugs), prescription databases provide information on all community prescriptions reimbursed by the NHS with drugs coded according to the Anatomical Therapeutic Chemical (ATC) classification system[16]. Hospital discharge records include information about primary diagnoses and up to five co-existing conditions, performed procedures, and in-hospital death. All diagnoses are coded according to the International Classification of Disease, Ninth Revision (ICD-9 CM) [17]. Civil registry provides information on age, sex, and death or migration.

The reliability of data sources and their linkage to produce epidemiological information have been previously described[18,19]. All security and protection measures for data from patients were performed according to the national law[20]. Data were obtained from the regional health authority in Puglia, Italy, providing data on all residents and were not generated or collected for this study. Data protection was ensured by the Healthcare Agency of Puglia. All data were anonymized prior to being accessed by the authors and none of the authors were involved in data anonymization. In Italy no ethical approval is required for aggregated-anonymous data.

### Baseline risk factors

Data from January 1^st^ 2003 to December 31^st^ 2010 were used to develop and validate the DDCI. A fixed cohort of all residents at 01/01/2004 and aged 40 years or above was identified from the civil registry of the Puglia region. Index date was represented by January 1^st^ 2004 for all citizens registered at the local health authority and alive at that time. The year of observation prior to the index date was used to define baseline characteristics. Patients were followed up from their index date to the earliest of death, migration, or the end of the study period. Prescription patterns indicative of chronic diseases (PPCD) and chronic exposure to drugs were derived from prescription databases.

Charlson Comorbidity Index (CCI) was calculated based on the diagnoses contained in hospital discharge databases.

### Outcome variables

The main outcome was overall mortality. The first unplanned hospital admission occurred after the index date, hospital readmissions, and 1-year mortality were considered as secondary outcomes. Overall survival was defined as the time between index date and death. For subjects who did not die, survival time was censored at the end of follow-up period or the date of leaving the region. The time horizon for risk prediction was set at 7 years.

### Study design

**Fig 1:**
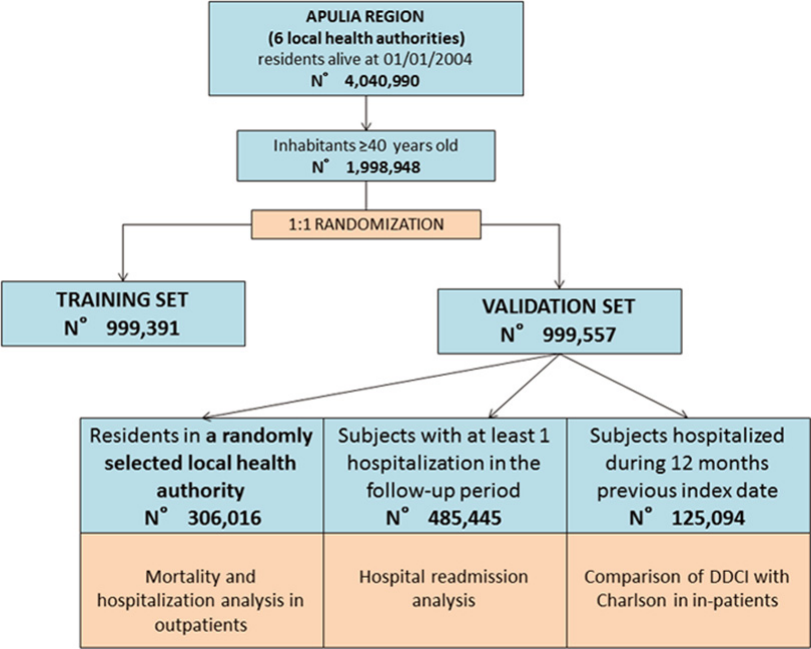
Flow-chart of the study.

To control the accuracy of predictions and to increase the reliability of all statistical analyses, the whole population was divided into 2 random samples (Fig 1):

- Training set, including 50% of subjects, in which different PPCDs and drug classes were included in a predictive model to develop the DDCI;
- Validation set, including the other half of the population, in which the predictive properties of the in DDCI were confirmed.

In addition, two cohorts were selected from the validation set:

1. a first cohort of randomly selected residents, in which the discrimination and reclassification power of DDCI were assessed;
2. a second cohort of all subjects hospitalized during 2003, in which the performance of DDCI was compared to that of the Charlson Comorbidity Index.

### Model building and statistical analysis

Patients’ baseline characteristics are reported as frequency (percentage) and mean±standard deviation (SD). A multivariate Cox proportional hazard regression model including all the PPCDs and drugs to which patients were chronically exposed at baseline was performed to identify predictors of overall mortality. All drug classes significantly associated with mortality risk were included in the final model. A weight assigned to each drug class was derived from regression coefficient value divided by 0.3; the value obtained was rounded to the nearest integer, as proposed by Gagne et al. [21]. The overall sum of weights determined the score of DDCI.

Overall mortality and time-to-first unplanned hospitalization analyses were performed using multivariate Cox proportional hazards regression models, and risks were reported as hazard ratios (HRs) with their 95% confidence interval (95% CI). Survival curves and probabilities were reported according to the Kaplan-Meier method. We evaluated the performance of the score in terms of discrimination and reclassification, with mortality and first unplanned hospitalization as outcomes. A model including age and sex was considered the reference model to which DDCI was added. In hospitalized subjects selected from the validation sample the same age and sex based reference model was used. Models adding separately the Charlson Comorbidity (CCI) index or the DDCI were tested. The final model included age, sex and both CCI and DDCI. Accurate predictions discriminate between those with and those without the outcome. Discrimination power was assessed by estimating survival C index with their 95% CI^22^. The reclassification extends the concept of discrimination by evaluating separately the subjects with and without outcome. The interpretation is opposite for subjects with and without the outcome. The proportion of events correctly reclassified and not-events correctly reclassified are summed. This sum was labeled as Net Reclassification Improvement (NRI) [22,23]. Subsequently, indices of discrimination and reclassification of the models were compared with the reference (adjusted only for age and sex). Readmissions to hospital were estimated as incidence rates (IRs; number of hospital readmissions per person years). A Poisson regression model was applied to estimate incidence rate ratios (IRRs) for individuals in the different DDCI score classes, taking the lowest class as the reference category. All statistical analyses were performed using SAS Software Release 9.4 (SAS Institute, Cary, NC).

## Results

Overall, a cohort of 1,998,948 subjects aged > = 40 years (mean age 60.17 ± 13.57 years, males 46.29%) was identified. The mean follow-up period was of 6.62±1.28 years, 12.53% had experienced at least 1 hospitalization for any cause and 76.25% had at least one drug prescription in the 12 months prior the index date. The training and the validation data sets included 999,391 and 999,557 subjects, respectively (Fig 1). During 7 years 106,664 deaths were registered in the training set and 106,590 in the validation set, corresponding to a cumulative mortality proportion of 10.67% and 10.66%, respectively. There were no statistically significant differences between the two groups in terms of age, gender, previous hospitalization, chronic exposure to the different drug types, and mortality rates (Table 1).

**Table 1:** Demographic and clinical characteristics of training and validation cohorts.

Characteristics	Training set	Validation set
N	999391	999557
Gender (M), n (%)	462816 (46.31)	462562 (46.28)
Age (y), mean±SD	60.17±13.58	60.17±13.57
Number of previous hospitalizations, mean±SD	0.19±0.62	0.19±0.62
Individuals with at least 1 previous hospitalization, n (%)	125356 (12.54)	125094 (12.51)
Individuals with at least 1 hospitalization in the follow-up period, n (%)	485378 (48.57)	485445 (48.57)
Individuals alive at the end of the follow-up period, n (%)	892727 (89.33)	892967 (89.34)
Medication use, n (%)		
Antiarrhythmics	10310 (1.03)	10202 (1.02)
Immunosuppressants	1917 (0.19)	1919 (0.19)
Platelet aggregation inhibitors	82487 (8.25)	82241 (8.23)
Parenteral anticoagulants	6540 (0.65)	6585 (0.66)
Oral anticoagulants	10364 (1.04)	10094 (1.01)
Antineoplastic agents	12153 (1.22)	12252 (1.23)
Inhaled bronchodilators	37316 (3.73)	37372 (3.74)
Drugs for arterial hypertension	294323 (29.45)	293631 (29.38)
Antihyperglycemic therapy	78998 (7.90)	78707 (7.87)
Drugs for hypertensive heart disease	51143 (5.12)	50991 (5.10)
Drugs for acid related disorders	202645 (20.28)	202807 (20.29)
Lipid modifying agents	67518 (6.76)	67691 (6.77)
Nonsteroidal anti-inflammatory drugs	179434 (17.95)	178948 (17.90)
Systemic Corticosteroids	16013 (1.60)	16058(1.61)
Opioids	516 (0.05)	550 (0.06)
Anti-Parkinson drugs	6696 (0.67)	6606 (0.66)
Antipsychotics	12120 (1.21)	12115 (1.21)
Anti-dementia drugs	2138 (0.21)	2145 (0.21)
Antidepressants	32987 (3.30)	32797 (3.28)

### Development of DDCI in training sample

In the training set, time-to-death analysis was used to assess weights of different PPCDs and drug classes. All the classes of drugs not significantly associated with overall mortality were excluded from the final model. Among the drug classes included in the final model, some (such as “antidepressants” and “nonsteroidal anti-inflammatory drugs”) showed a poor contribution to mortality risk, totalizing a score of 0. Opioids appeared to be the drugs more related to mortality risk. The use of lipid modifying agents and immunosuppressants was associated with a risk of death lower than the reference category, represented by individuals not taking any of the drugs considered, and therefore a negative score was assigned to them. The regression coefficient values calculated on overall mortality and the weight assigned to each drug class of the best-in-class model are shown in Table 2. DDCI was obtained through the algebraic sum of the weights of the different drug classes. The score of DDCI ranges between -3 and 33. Due to the small number of cases, subjects with a negative score of DDCI were incorporated into the lowest risk class, represented by individuals with a score of 0; similarly, the upper class contains values of 11 or greater; therefore, 12 classes of DDCI score were eventually identified.

**Table 2:** Assignment of weights in the construction of DDCI through a time-to-death multivariate Cox proportional hazards regression.

Drug classes	Definition	Regression coefficient	Hazard Ratio	CI	Weight
Antiarrhythmics	At least 3 packages of drugs with ATC codes C01B within 12 months	0.418	1.52	1.47–1.57	1
Immunosuppressants	At least 3 packages of drugs with ATC codes L04 within 12 months	-0.368	0.69	0.62–0.78	-1
Platelet aggregation inhibitors	At least 3 packages of drugs with ATC codes B01AC within 12 months	0.532	1.70	1.67–1.73	2
Parenteral anticoagulants	At least 3 packages of drugs with ATC codes B01AB or B01AX within 12 months	0.435	1.55	1.48–1.62	1
Oral anticoagulants	At least 3 packages of drugs with ATC codes B01AA within 12 months	0.368	1.45	1.39–1.50	1
Antineoplastic agents^26^	at least 3 packages of drugs with ATC codes L01 within 12 months	0.880	2.41	2.33–2.49	3
Inhaled bronchodilators^17-18^	at least 3 packages of drugs with ATC code R03A, R03BB, R03DA within 12 months	0.739	2.09	2.05–2.14	2
Drugs for arterial hypertension^16^	at least 3 packages of drugs with ATC codes C02A, C02C, C02LA, C02LB, C03A, C03BA, C03EA01, C07AA, C07AB (excluded C07AB09), C07AG, C07BB, C07C, C08, C09AA, C09BA, C09CA, C09DA within 12 months	0.390	1.48	1.46–1.50	1
Antihyperglycemic therapy^19^	at least 2 packages of drugs with ATC code A10 within 12 months	0.530	1.70	1.67–1.73	2
Drugs for hypertensive heart disease^15-17^	presence within 45 days of: -any combination of drugs with ATC code C01AA05, C03CA01, C03DA01, C07AG02, C07AB07, C07AB03, C09; -at least 2 prescriptions of drugs with ATC code C01AA05;	0.831	2.30	2.26–2.34	3
Drugs for acid related disorders	At least 3 packages of drugs with ATC codes A02 within 12 months	0.289	1.34	1.32–1.35	1
Lipid modifying agents^25^	at least 3 packages of drugs with ATC codes C10 within 12 months	-0.560	0.57	0.56–0.58	-2
Nonsteroidal anti-inflammatory drugs	At least 3 packages of drugs with ATC codes M01 within 12 months	0.087	1.09	1.08–1.11	0
Systemic Corticosteroids	At least 3 packages of drugs with ATC codes H02AB within 12 months	0.464	1.59	1.54–1.64	2
Opioids	At least 3 packages of drugs with ATC codes N02A (other than Codein and Tramadol) within 12 months	1.672	5.32	4.81–5.88	6
Anti-Parkinson drugs	At least 1 package of drugs with ATC codes N04 within 12 months	1.108	3.03	2.93–3.14	4
Antipsychotics	At least 1 package of drugs with ATC codes N05A within 12 months	0.841	2.32	2.24–2.40	3
Anti-dementia drugs	At least 1 package of drugs with ATC codes N06A within 12 months	1.130	3.10	2.92–3.29	4
Antidepressants	At least 1 package of drugs with ATC codes N06D within 12 months	0.087	1.09	1.06–1.11	0

### Results of DDCI application on validation set

**Fig 2:**
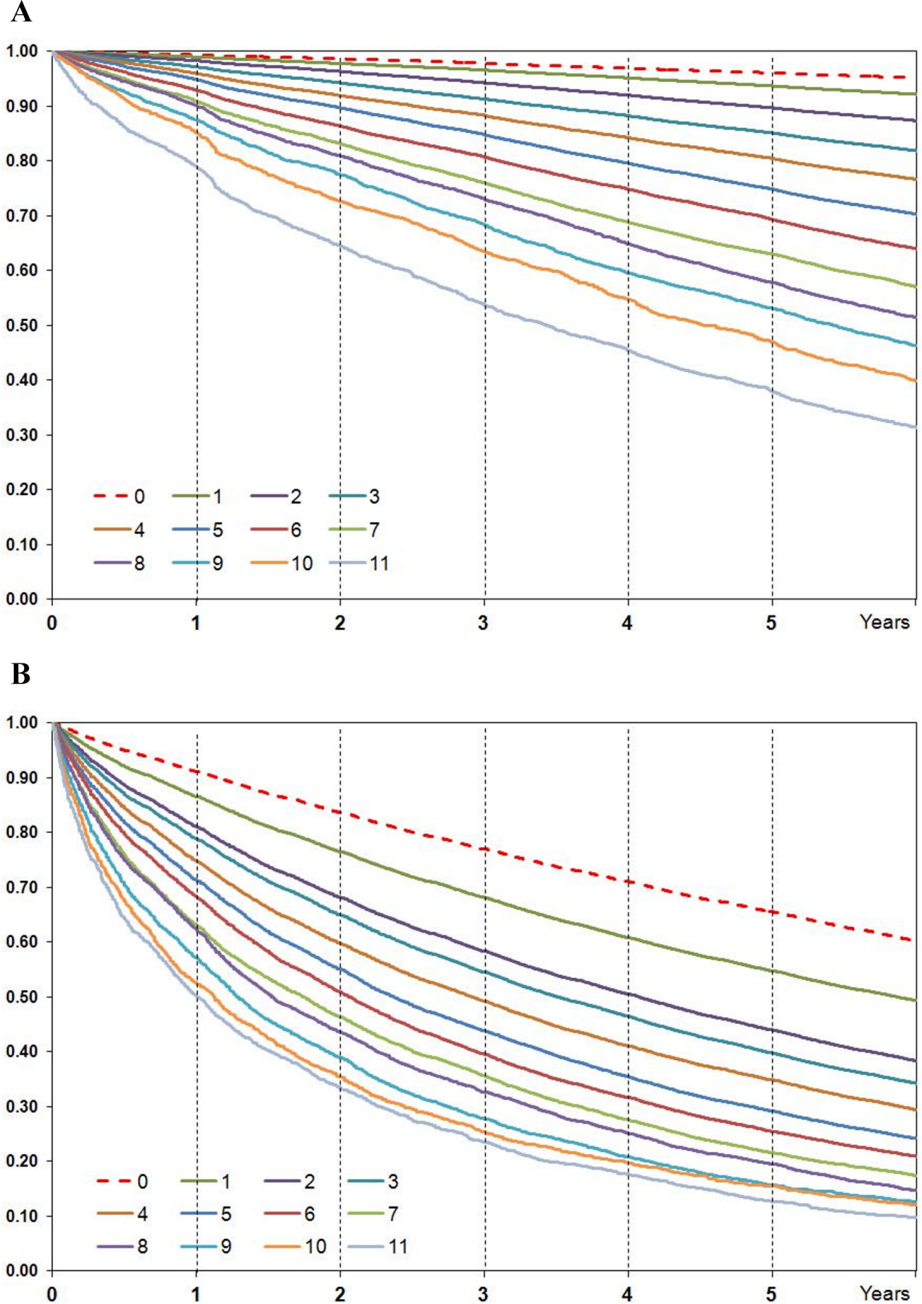
Kaplan-Meier curves according to DDCI score value. Panel A: overall survival; Panel B: time to first hospitalization.

A statistically significant increase in hazard ratios values with the increase in DDCI score was documented in the validation cohort, closely reproducing the results obtained in the training sample (Table 3). In particular, overall mortality was below 5% in the lowest risk group, while it exceeded 70% in the highest risk group. Survival curves with Kaplan-Meier method according to DDCI score values are reported in Fig 2 Panel A.

**Table 3:** Hazard Ratios for overall mortality adjusted by age and sex, according to DDCI.

	TRAINING SAMPLE		VALIDATION SAMPLE	
	HR (95% CI)	p	HR (95% CI)	P
DDCI≤0	1.00		1.00	
DDCI = 1	0.98 (0.96–0.99)	< .0001	0.98 (0.96–1.00)	0.04
DDCI = 2	1.27 (1.24–1.30)	< .0001	1.24 (1.21–1.26)	< .0001
DDCI = 3	1.61 (1.58–1.65)	< .0001	1.61 (1.58–1.65)	< .0001
DDCI = 4	1.93 (1.89–1.98)	< .0001	1.93 (1.88–1.97)	< .0001
DDCI = 5	2.26 (2.20–2.32)	< .0001	2.29 (2.23–2.35)	< .0001
DDCI = 6	2.69 (2.62–2.76)	< .0001	2.70 (2.62–2.77)	< .0001
DDCI = 7	3.18 (3.09–3.28)	< .0001	3.25 (3.15–3.34)	< .0001
DDCI = 8	3.68 (3.55–3.82)	< .0001	3.60 (3.47–3.74)	< .0001
DDCI = 9	4.32 (4.15–4.50)	< .0001	4.15 (3.98–4.32)	< .0001
DDCI = 10	4.70 (4.43–4.98)	< .0001	4.57 (4.32–4.84)	< .0001
DDCI≥11	6.16 (5.87–6.45)	< .0001	5.89 (5.62–6.17)	< .0001

DDCI was also able to predict time to first unplanned hospitalization; Kaplan-Meier curve showed a progressive increase in unplanned hospital admission risk with increasing DDCI score (Fig 2, Panel B). Among the 485,445 subjects with a first unplanned hospitalization in the follow-up period, the DDCI score also predicted hospital readmission. The age and gender adjusted risk of hospital readmission during the period of observation increased with the DDCI score, and the highest risk group had an incidence rate ratio of hospital readmission per person-year equal to 5.62 (5.48–5.66) when compared to the lowest risk class (Table 4).

**Table 4:** Incidence rates (IR) per person-years of total hospital readmissions in the validation set and incidence rate ratios (IRR) according to DDCI score (the lowest class is the reference category).

Subgroups	IR	IRR (95% CI)
DDCI≤0	0.50 (0.49–0.50)	1.00
DDCI = 1	0.68 (0.68–0.69)	1.38 (1.36–1.39)
DDCI = 2	0.92 (0.91–0.93)	1.86 (1.84–1.88)
DDCI = 3	1.14 (1.13–1.16)	2.31 (2.28–2.34)
DDCI = 4	1.31 (1.29–1.32)	2.63 (2.60–2.66)
DDCI = 5	1.51 (1.49–1.53)	3.04 (3.00–3.08)
DDCI = 6	1.77 (1.75–1.79)	3.56 (3.51–3.62)
DDCI = 7	1.92 (1.89–1.95)	3.87 (3.81–3.93)
DDCI = 8	2.23 (2.19–2.27)	4.50 (4.41–4.59)
DDCI = 9	2.70 (2.65–2.76)	5.45 (5.34–5.56)
DDCI = 10	2.63 (2.55–2.71)	5.30 (5.14–5.46)
DDCI≥11	2.79 (2.72–2.86)	5.62 (5.48–5.66)

The evaluation of accuracy, discrimination and reclassification capacity of DDCI was performed for the residents of the largest local health authority (N = 306,016) (Table 5, Model A). As for 1-year mortality the estimated survival C index pointed out that in the model with age, sex and DDCI the discrimination power was slightly better than the model with only age and sex (0.851 [0.846–0.856], 0.815 [0.809–0.820], respectively). In other words, age, sex and DDCI altogether had a probability of 85.1% to predict 1-yearmortality. As for overall mortality the gain in the discrimination power by adding DDCI was slightly lower (0.835 [0.83–0.837]) than the analogous model relative to the 1-year mortality analysis. A slight improvement in term of discrimination masked a much greater improvement in terms of reclassification. In fact, the 1-year mortality analysis showed that the model including DDCI was much more accurate than the reference model (NRI = 0.698 [0.673–0.725]) with a 16.3% of events correctly reclassified and 53.5% of non-events correctly reclassified. As for the overall mortality, despite a reclassification improvement (NRI = 0.586 [0.571–0.600]), a decrease of 7% of events correctly reclassified was shown. DDCI performed better in the correct reclassification of non-events, demonstrating an excellent capacity in the identification of the low risk population. DDCI showed a better performance in term of events correctly reclassified for one-year mortality compared to overall mortality and first unplanned hospitalization (with respect to overall mortality and first unplanned hospitalization).

**Table 5:** Discrimination and reclassification analysis.

Outcomes	Survival C-Index (CI)	NRI (CI)	Proportion of events correctly reclassified	Proportion of non-events correctly reclassified
Model A				
1-Year Mortality	0.851 (0.846–0.856) Vs. 0.815 (0.809–0.820) ^$^	0.698 (0.673–0.725)	0.1634	0.5349
Overall Mortality	0.835 (0.833–0.837) Vs. 0.815 (0.813–0.817) ^$^	0.586 (0.571–0.600)	-0.070	0.6563
1^st^-Unplanned Hospitalization	0.584 (0.582–0.586) Vs. 0.585 (0.583–0.587) ^$^	0.301 (0.290–0.310)	-0.2341	0.5351
Model B				
1-Year Mortality	0.763 (0.757–0.768) Vs. 0.715 (0.710–0.721) ^$^	0.531 (0.506–0.552)	0.2660	0.2652
Overall Mortality	0.774 (0.771–0.776) Vs. 0.740 (0.737–0.742) ^$^	0.525 (0.509–0.539)	0.1475	0.3770
1st-Unplanned Hospitalization	0.606 (0.603–0.608) Vs. 0.598 (0.596–0.600)$	0.403 (0.388–0.421)	0.0615	0.3419
Model C				
1-Year Mortality	0.798 (0.793–0.802) Vs. 0.715 (0.710–0.721) ^$^	0.528 (0.501–0.553)	0.1403	0.3880
Overall Mortality	0.785 (0.783–0.787) Vs. 0.740 (0.737–0.742) ^$^	0.739 (0.727–0.750)	0.4899	0.2487
1st-Unplanned Hospitalization	0.618 (0.616–0.620) Vs. 0.598 (0.596–0.600)^$^	0.505 (0.487–0.517)	0.0463	0.4586
Model D				
1-Year Mortality	0.810 (0.806–0.815) Vs. 0.798 (0.793–0.802) *	0.263 (0.239–0.284)	0.1179	0.1451
Overall Mortality	0.795 (0.793–0.797) Vs. 0.785 (0.783–0.787) *	0.342 (0.329–0.357)	0.0544	0.2874
1st-Unplanned Hospitalization	0.620 (0.617–0.622) Vs. 0.618 (0.616–0.620) *	0.275 (0.257–0.259)	-0.0086	0.2832

Model A: Comparison between the model including DDCI and the model with only age and sex; results in one randomly selected local health authority (N = 306,016)

Model B: Comparison between the model including DDCI and the model with only age and sex; results in the cohort of hospitalized patients (N = 125,094)

Model C: Comparison between the model including CCI and the model with only age and sex; results in the cohort of hospitalized patients (N = 125,094)

Model D: Comparison between the model including DDCI and the model with age, sex and CCI; results in the cohort of hospitalized patients (N = 125,094)

^$^ Age, sex (reference model)

* Age, sex and CCI (reference model)

Positive values of the Proportion of events correctly reclassified or of the Proportion of non-events correctly reclassified indicate a improvement in reclassification; negative values a worsening in reclassification. Net Reclassification Improvement (NRI) is the algebraic sum of these two proportions and positive values of NRI indicate and improvement in overall reclassification capacity.

### Comparison between DDCI and Charlson Comorbidity Index

In the validation data set, 125,094 citizens had had at least one hospitalization during the 12 months prior the index-date. The clinical risk for these subjects was assessed through the application of the Charlson Comorbidity Index and the DDCI. When compared to the reference model (age and sex), both DDCI (Table 5 - Model B) and CCI (Table 5 - Model C) markedly improved prediction. In particular, CCI showed a better survival C index on short and long-term outcomes and a better performance than DCCI in reclassification on overall-mortality.

In the final model (Table 5 - Model D), the 1-year-mortality analysis showed that the model including also DDCI was more accurate than the model with age, sex and CCI (NRI = 0.263 [0.239–0.284]) with 11.8% of events correctly reclassified and 14.5% of non-events correctly reclassified. The overall mortality analysis showed that the model including also DDCI was more accurate than the model with age, sex and CCI (NRI = 0.342 [0.329–0.357]), with 5.4% of events correctly reclassified and 28.7% of non-events correctly reclassified

As for the prediction of first unplanned hospitalization, both DDCI and CCI showed a better performance when compared to the reference model, with a further improvement when used simultaneously.

## Discussion

### Main findings

Our study shows that a simple score, based on drug prescriptions as proxies of chronic conditions, is able to stratify the risk of the general population in terms of short and long term mortality, unplanned hospital admission and readmission. In hospitalized individuals, the performance of the DDCI score was similar to that of the Charlson index. When used in combination with the Charlson index, the DCCI significantly improved the prediction, thus representing an added value even in the presence of clinical information. Since the DCCI score is solely based on drug prescriptions, it allows the risk stratification of entire populations, without the need for clinical data, hardly available at the population level.

### Comparison with existing data

Many clinical risk models are been proposed to predict death or unplanned hospitalization risk^4^, using different approaches:

- “threshold modelling” [24,25], that has proven to be more accurate when used within a specific clinical context than within a general population[26–28];
- “clinical knowledge”, in which physicians may be able to identify current high risk patients. Exportability of these models presents limitations due to problems in standardizing clinical judgements of different physicians in predicting citizens that may become high risk patients[29];
- “predictive modeling”, that uses regression models and appears to be more effective than other techniques[4].

There are a variety of predictive tools used for the identification of high risk patients through the integration of clinical, laboratory, functional, socio-familiar, and care variables into statistical predictive models[30–37]. Obtaining all this information in large populations requires an expensive ad-hoc clinical data collection, making this approach unfeasible in many instances. In alternative, administrative health databases can be used to obtain algorithms useful to forecast readmission[38,39] and reduce in-care cost[7,40]. Validated comorbidity indexes have also been applied on hospitalization data to predict the risk of death or readmission[8,9]. These models do not permit to define the out-patient risk profile[35], unless full clinical documentation is available. More complex predictive models were obtained to overcome this limitation through the integration of several data-sources[41–44], including outpatients, accident and emergency, electronic clinical data from general practitioners[35], socio-economic data[45], and community dispensed prescriptions. Unfortunately, the different data-bases required are not always available and/or standardized. Our data show that a much simpler scoring system, solely based on drug prescriptions, can accurately predict one-year and long-term mortality, as well as the risk of unplanned hospitalization and hospital readmission, thus overcoming many of the limitations of previous predictive instruments. These outcomes are the most important determinant of “frailty” [46,47] and a standardized tool able to stratify the clinical risk profile of patients is a priority in many healthcare settings[48].

Some predictive models derived by prescribed drug registers are been experienced[49]; nevertheless, the drug-based comorbidity scores previously developed address other issues or show some limitations: 1) the small sample[50]; 2) the arbitrary choices of the class of drugs to be included in the model[50,51]; 3) the objective of the tool circumscribed to the prediction of health care costs[52]; 4) the poor ability to predict mortality[53]; 5) the lack of a sufficient spatial and temporal coverage to perform the analysis[50–52].

### Implications for clinical practice

The DDCI score can be used to help policy planners to identify at the population level those individuals showing a higher likelihood of intensive resource utilization. A focused, proactive approach targeted to these individuals may avoid that their health deteriorate to such a point that they need to be hospitalized, with positive implications also in terms of costs of care. As an example, several studies suggest that focused care after discharge can improve post-discharge outcomes and avoid readmission[54–56].

Since hospital admission, particularly early readmissions can be considered to be an indicator of poor quality of care (i.e. unsuccessful discharge processes or inadequate social care[57]), the DDCI score can also be used as a case-mix measure to compare the performance of different structures/health districts.

The availability of a tool capable to capture the level of clinical complexity of patients could assist physician in their everyday practice, allowing to make more objective their clinical judgments. The possibility of an uniform assessment of the complexity of the patients could also facilitate the definition of guidelines and care models centered on patients with multiple morbidities and carrying a high risk of unfavorable outcomes.

Moreover, since the last year of life is characterized by high healthcare costs, the identification of individuals at high risk of short-term death can be of great significance to health providers and insurers[58].

### Strengths and limitations

The major strength of the DDCI score is its reliance on a single source of administrative data based on the ATC coding system, widely utilized in many countries. For this reasons the DDCI can be applied in all the healthcare contexts in which there is a lack of clinical data; it can be easily applicable at population level, requiring only the availability of data on drug prescriptions.

The study also has limitations. First the application of DDCI at the population level is not generalizable outside of comprehensive health networks or all-payer administrative datasets. Nevertheless, the score can be used in any database containing drug prescriptions (for example general practitioners databases) or within studies to stratify the clinical risk profile of specific cohorts. It can be also used as a case-mix adjustment measure whenever clinical data needed to calculate CCI are not available.

Second, in our cohort, the use of lipid modifying agents and immunosuppressants was associated with a risk of death lower than the reference category. These results could appear counterintuitive. However, the use of lipid lowering drugs was very limited (6.7% of the population). We can thus hypothesize that within the reference group represented by individuals not treated with any of the drug classes considered there were also individuals candidate to lipid lowering treatment but not receiving the drug. This can be responsible for a higher risk of death in the reference category as compared to people treated with statins. As for immunosuppressants, we do not have an obvious explanation. Nevertheless, it should be noted that this class represented less than 0.2% of the whole cohort, making its contribution to the scoring almost irrelevant. Third, it should be emphasized that the scoring system can underestimate the risk in some individuals, due to the lack of information on those drugs, particularly cancer drugs, dispensed at the hospital. Furthermore, it reflects the risk of the Italian population, and its testing in other healthcare systems is warranted.

Finally, DDCI was not externally validated, but the random split in 2 equally large dataset (training and validation set) of a whole regional population of approximately 2 million of people constitutes a reliable methodology for the validation. Anyway, further studies are needed to evaluate the performance of DDCI as compared to CCI on an external outpatients population.

## Conclusions

We developed and validated a prognostic index derived from prescription data, able to stratify the entire population into homogeneous risk groups. DDCI can represent a useful tool for risk-adjustment and for policy planning, as well as an instrument for the identification of patients needing a focused approach in the everyday practice. Its use can thus help improving the quality of care and optimize resource allocation.
